# An Important Local Government Appointment

**Published:** 1912-12-14

**Authors:** 


### An Important Local Government Appointment.

### MISS LUCY W. WAMSLEY.

MISS WAMSLEY, after four years' hard and most successful work at the Royal Victoria Infirmary, Newcastle-on-Tyne, has been selected by the Local Government Board to fill the important post of one of the women inspectors. We give a brief account of her work at Newcastle and offer her our hearty congratulations. We hope she may have the best of health to follow out zealously for many years to come the new work with which she has been entrusted.

Miss Wamsley came as matron to the Royal Victoria Infirmary in 1908, eighteen months after the opening of the new buildings. Her predecessor, Miss McCall Anderson, who came shortly after the opening of the new buildings, had done a great deal to organise the staff, nursing and domestic; she, indeed, had borne the brunt of the move, but when she left there was still a great deal to do and many difficulties to be overcome. The full number of beds were not actually in use until some months after Miss Wamsley took over the post. Those who understand the difficulties connected with the nursing of a large hospital will appreciate what it means to double a large nursing staff. At the old infirmary the staff of nurses was between seventy and eighty. The present staff of nurses under Miss Wamsley is between 140 and 150. In addition to the increase in the actual numbers many reforms had to be carried out in the training, the teaching, the examinations, etc., of the nurses. The nurses are instructed by paid lecturers, they are taught in the intervals between lectures by one of the senior sisters; classes are arranged for sick-room cookery; examinations are held by outside examiners and annual prizes to the value of between £35 and £40 are given to those who do best in the examinations. Probationers in their first year are now paid a salary and the salaries of the nurses generally have been increased. The course of training has been changed from three to four years, certificates being granted at the end of the third year, the nurse being under an obligation to serve the hospital for a fourth year. Miss Wamsley in the time at her disposal has raised the nursing at the Royal Victoria Infirmary until it now ranks as one of the great training schools in the Kingdom. She has not had time to establish a preliminary training school for nurses, nor a private institution; these she has left for her successor. In addition to her work connected with the nursing, Miss Wamsley has formed and had under her control a large domestic staff in laundry, in kitchen, and in wards and nurses' home. She has, indeed, admirably carried out the work so well begun by Miss McCall Anderson, and the committee at the annual prize-giving of the nurses held on December 6 presented her with an illuminated testimonial in recognition of her services and wishing her health and happiness in the work under the Local Government Board which she is to take up in January 1913.

